# Osteopathic research: elephants, enigmas, and evidence

**DOI:** 10.1186/1750-4732-1-7

**Published:** 2007-02-08

**Authors:** John C Licciardone

**Affiliations:** 1 Osteopathic Research Center, University of North Texas Health Science Center-Texas College of Osteopathic Medicine, Fort Worth, TX 76107, USA

## Abstract

**Background:**

The growth and acceptance of osteopathic physicians as conventional medical practitioners in the United States has also raised questions about the distinctive aspects of osteopathic medicine. Although the use of osteopathic manipulative treatment (OMT) and a focus on primary care are most often cited as rationales for the uniqueness of osteopathic medicine, an osteopathic professional identity remains enigmatic.

**Discussion:**

The fledgling basic osteopathic research efforts of the early and mid-twentieth century have not been sustained and expanded over time. Thus, there is presently a scarcity of basic mechanistic and translational research that can be considered to be uniquely osteopathic. To be sure, there have been advances in osteopathic clinical trials, particularly those involving OMT for low back pain. Meta-analysis of these low back pain trials has provided evidence that: (1) OMT affords greater pain reduction than active or placebo control treatments; (2) the effects of OMT are comparable regardless of whether treatment is provided by fully-licensed osteopathic physicians in the United States or by osteopaths in the United Kingdom; and (3) the effects of OMT increase over time. However, much more clinical research remains to be done. The planning and implementation of a large longitudinal study of the natural history and epidemiology of somatic dysfunction, including an OMT component, represents a much-needed step forward. Osteopathic medicine's use of OMT and its focus on primary care are not mutually exclusive aspects of its uniqueness. The intersection of these fundamental aspects of osteopathic medicine suggests that the profession may successfully adopt a generic strategy of "focused differentiation" to attain a competitive advantage in the health care arena. While there are both requisite demands and risks for the osteopathic profession in adopting such a strategy, these are reasonable in relation to the potential rewards to be attained. To help promote an osteopathic identity, "*omtology*" and its derivative terms are recommended in referring to the study of OMT.

**Conclusion:**

The osteopathic profession should adopt a coherent strategy for developing and promoting its identity. Failure to do so will likely ensure that osteopathic medicine remains "stuck in the middle."

## Background

Osteopathy experienced substantial evolution and growth during the twentieth century. The emergence and acceptance of osteopathic medicine as a "mainstream" or "conventional" medical system appears to afford unparalleled opportunities for the next generation of osteopathic physicians. Upon closer inspection, however, the triumphs of osteopathic medicine and its position on the American medical landscape may also herald a new challenge – that for its long-term survival. Increasingly, osteopathic physicians (DOs) are asked what makes them distinctive (i.e., different than allopathic physicians, or MDs). Inevitably the responses focus on two rationales – the use of osteopathic manipulative treatment (OMT) and an emphasis on primary care. The objective of this commentary is to review the history and current state of osteopathic research, within the overarching framework of contemporary medical practice with its emphasis on evidence-based medicine and the "business" of medicine, and to offer some suggestions on future directions for the osteopathic profession.

## Discussion

### The present need for research on osteopathic manipulative treatment

A.T. Still established the osteopathic philosophy based on anatomical and physiological principles. Prior to the rise of the pharmaceuticals industry, the growth of osteopathy was largely attributed to OMT and its presumed therapeutic benefits. The licensure of osteopathic physicians in the United States was *de facto *justification for OMT's place in their clinical armamentarium prior to the emergence of rigorous clinical trial methodologies.

However, several factors during the latter half of the twentieth century altered this paradigm. First, the growth of the pharmaceuticals industry and the regulatory need for demonstrating the safety and efficacy of new drugs led to the development of and reliance on randomized controlled trials. Second, traditional epidemiologic research methods developed for public health began to be applied to clinical populations. Hastened by the growing power and availability of computers, supporting software applications, and health-related databases, the field of clinical epidemiology blossomed and subsequently refined the tools for contemporary evidence-based medicine. Third, with the requisite methodologies and resources to collect and analyze clinical data widely available, government and other third-party payers increasingly demanded evidence not only of the safety and efficacy of clinical interventions, but also of their cost-effectiveness. Consequently, in response to these phenomena, there is a present need to demonstrate the safety, efficacy, and cost-effectiveness of OMT.

### Somatic dysfunction and its relationship to osteopathic manipulative treatment

Somatic dysfunction is defined as impaired or altered function of related components of the somatic (body framework) system: skeletal, arthrodial, and myofascial structures, and related vascular, lymphatic, and neural elements [1]. Given the presence of somatic dysfunction and its potential relationship to clinical or subclinical disease, it is reasonable to hypothesize that OMT may be a useful primary or complementary modality in a variety of clinical entities encountered by osteopathic physicians.

**Figure 1:**
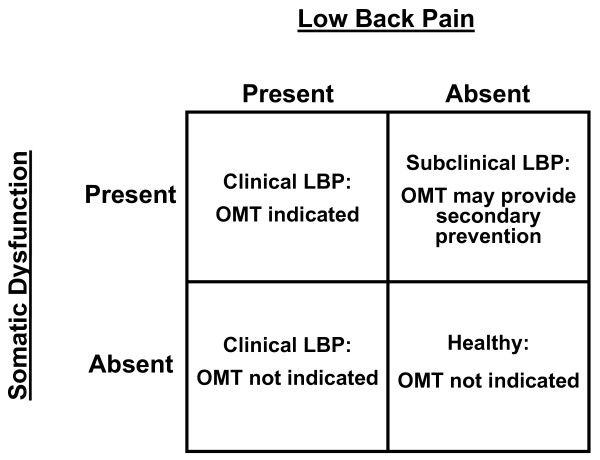
The utility of osteopathic manipulative treatment (OMT) according to the potential relationships between somatic dysfunction and low back pain (LBP).

The concept of somatic dysfunction raises some fundamental questions regarding its causal relationship to disease states and its responsiveness to OMT [2]. Is somatic dysfunction *sufficient *to cause a particular disease? Is somatic dysfunction *necessary *to cause a particular disease? The most likely and complex scenario occurs when the response to each of the previous questions is negative. For example, when considering low back pain as the "disease," this scenario leads to the 2 × 2 table presented in Figure 1. The following conclusions may thus be drawn: (1) OMT may not be indicated for all patients with low back pain; and (2) OMT may be useful in some patients with somatic dysfunction, but without low back pain. The first conclusion has important implications for establishing inclusion and exclusion criteria when designing clinical trials of OMT. The second conclusion suggests that OMT may be useful as a secondary preventive measure.

**Figure 2:**
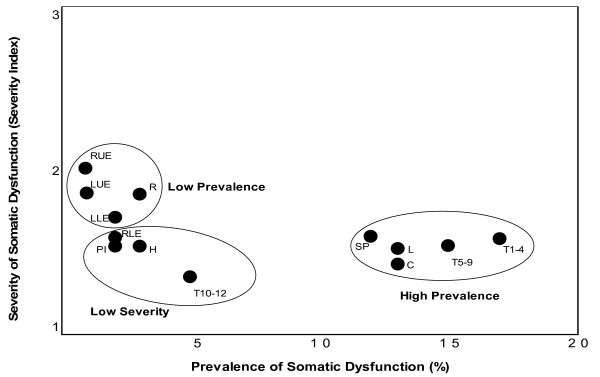
The burden of somatic dysfunction as a function of prevalence and severity. The anatomical regions are: C, cervical; H, head; L, lumbar; LLE, left lower extremity; LUE, left upper extremity; PI, pelvis/innominate; R, ribs; RLE, right lower extremity; RUE, right upper extremity; SP, sacrum/pelvis; T, thoracic.

The foregoing discussion highlights the need for research on the natural history and epidemiology of somatic dysfunction. Relatively little research has been performed on this integral aspect of osteopathic theory and practice. A retrospective analysis of family medicine patients attending university-based clinics was recently performed to begin addressing this issue [3]. This study measured the burden of somatic dysfunction at various anatomical regions as a function of prevalence and severity. As shown in Figure 2, using cluster analysis, three distinct groups emerged: (1) "high prevalence of somatic dysfunction"; (2) "low prevalence of somatic dysfunction"; and (3) "low severity of somatic dysfunction." It should be emphasized that the prevalence and severity of somatic dysfunction and, consequently, the burden of somatic dysfunction will vary with the methodological rigor and clinical population of a given study.

A large, long-term longitudinal study is urgently needed to extend our knowledge in this fundamental area. Such a study could not only address the natural history and epidemiology of somatic dysfunction, but also its response to OMT. Following a baseline osteopathic examination, the study would be facilitated by including a cohort of subjects followed by osteopathic physicians and another cohort followed by allopathic physicians. Because the latter do not diagnose or treat somatic dysfunction, the natural history of somatic dysfunction in the absence of OMT could be ethically and validly studied. Additionally, elegant case-control studies could be nested within this longitudinal design. The importance of this study to the osteopathic profession would not be overstated by referring to it as the "Osteopathic Framingham Study."

### Osteopathic manipulative treatment: the elephant in the room?

Some observers of osteopathic medicine criticize its continued promotion of OMT because of a perceived lack of evidence regarding its efficacy. One point of view is that belief in the efficacy of OMT is based more on faith than on evidence [4]. Certainly, osteopathic physicians should strive to demonstrate the efficacy of OMT using contemporary standards of evidence-based medicine whenever possible. On the other hand, how many routinely accepted non-pharmacological interventions, such as specific surgical procedures and psychotherapeutic interventions, can boast of undisputed efficacy demonstrated through large randomized controlled trials?

Important osteopathic clinical trials are being conducted and planned. Sponsors of these clinical trials include the National Institutes of Health, the Osteopathic Heritage Foundations, and other organizations. For example, the Osteopathic Research Center coordinates two major trials: the Multi-Center Osteopathic Pneumonia Study in the Elderly (MOPSE) (ClinicalTrials.gov identifier, NCT00258661) and a large clinical trial of OMT for chronic low back pain (ClinicalTrials.gov identifier, NCT00315120). The Osteopathic Research Center hopes to soon begin enrolling subjects in two other clinical trials. One trial involves the use of OMT for low back pain in active duty military personnel, while the other entails OMT to treat carpal tunnel syndrome. The Multi-center Osteopathic Otitis Media Study (MOMS), which will assess the efficacy of OMT for otitis media in children, is presently at an advanced developmental stage.

### The evidence for osteopathic manipulative treatment of low back pain

Not surprisingly, because of the historical preponderance of low back pain as a reason for visiting osteopathic physicians, osteopathic clinical trials have addressed low back pain more often than any other condition [5]. Thus, low back pain may be viewed as the prototypical condition for examining the present state of osteopathic clinical research. In 1994, the Agency for Health Care Policy and Research issued its far-reaching clinical practice guideline on acute low back problems in adults [6]. The recommendations on spinal manipulation are presented in Table 1. It is important to note that these recommendations reflect spinal manipulation generally, as practiced by a variety of practitioners such as chiropractors and physical therapists, and are not specific to osteopathic physicians. An updated evaluation of this guideline in 2000 did not report any important new evidence on spinal manipulation [7]. Similar guidelines for the management of low back pain in primary care have been developed internationally [8].

**Table 1:** Recommendations of the Agency for Health Care Policy and Research on spinal manipulation for low back problems in adults*

**Recommendation**	**Rating**	**Level of evidence**
Manipulation can be helpful for patients with acute low back problems without radiculopathy when used within the first month of symptoms.	B	Moderate research-based evidence
When findings suggest progressive or severe neurologic deficits, an appropriate diagnostic assessment to rule out serious neurologic conditions is indicated before beginning manipulation therapy.	D	Panel interpretation of information that did not meet inclusion criteria as research-based evidence
There is insufficient evidence to recommend manipulation for patients with radiculopathy.	C	Limited research-based evidence
A trial of manipulation in patients without radiculopathy with symptoms longer than one month is probably safe, but efficacy is unproven.	C	Limited research-based evidence
If manipulation has not resulted in symptomatic improvement that allows increased function after one month of treatment, manipulation therapy should be stopped and the patient re-evaluated.	D	Panel interpretation of information that did not meet inclusion criteria as research-based evidence

*Based on reference 6

**Figure 3:**
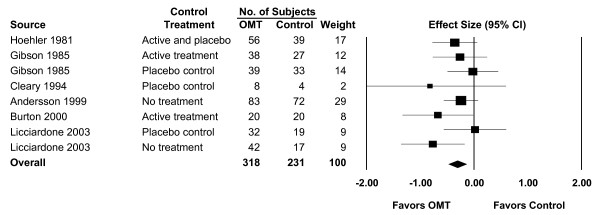
Meta-analysis results for osteopathic manipulative treatment (OMT) of low back pain. The overall effect size was -0.30 (95% confidence interval [CI], -0.47 – -0.13; P = .001). Source citations are available in reference 5.

The clinical trials involving OMT for low back pain have been published in such high-impact journals as *The New England Journal of Medicine *[9], *The Lancet *[10], the *Journal of the American Medical Association *[11], and *Spine *[12]. The three major osteopathic clinical trials involving low back pain performed in the United States have been previously reviewed and summarized [13]. The overall results of a recent systematic review and meta-analysis of OMT for low back pain [5] are shown in Figure 3. The mean effect size (ES) for OMT was -0.30 (95% confidence interval [CI], -0.47 – -0.13; P = .001), indicating a statistically significant and clinically important reduction in low back pain with OMT. A comprehensive series of subgroup and sensitivity analyses were also performed as part of the meta-analysis. These demonstrated that: (1) OMT provided greater pain reduction than active or placebo control treatments (ES, -0.26; 95% CI, -0.48 – -0.05; P = .02); (2) the effects of OMT were comparable regardless of whether treatment was provided by fully-licensed osteopathic physicians in the United States (ES, -0.31; 95% CI, -0.52 – -0.10; P = .004) or by osteopaths in the United Kingdom (ES, -0.29; 95% CI, -0.58 – -0.00; P = .05); and (3) the effects of OMT increased over time (ES, -0.28; 95% CI, -0.51 – -0.06; P = .02 for short-term treatment [less than one month]; ES, -0.33; 95% CI, -0.51 – -0.15; P < .001 for intermediate-term treatment [one month to less than three months]; ES, -0.40; 95% CI, -0.74 – -0.05; P = .03 for long-term treatment [three months or longer]).

Several important implications derive from these findings. The pain-reducing effects of OMT are significantly greater than those of a placebo or sham treatment. In fact, by comparison with published data, the pain reduction afforded by OMT is comparable to that of non-steroidal anti-inflammatory drugs, including cyclo-oxygenase-2 inhibitors [14], and may potentially last longer than these drugs [15]. With the adverse events known to be associated with non-steroidal anti-inflammatory drugs, and more intense scrutiny of the safety profile of cyclo-oxygenase-2 inhibitors, OMT may offer an attractive alternative to such drugs in many patients with low back pain, particularly those patients requiring ongoing treatment of chronic pain. Finally, for planning purposes, it is important to recognize that previous osteopathic clinical trials have generally been inadequately powered to detect outcomes at the threshold of clinical relevance.

### Osteopathic enigmas

Webster's Thesaurus lists the following synonyms for the term "enigma": puzzle, riddle, question, perplexity, conundrum, mystery, secret, and hidden meaning. A cluster of vaginal adenocarcinoma cases among young women at a Boston hospital between 1966 and 1969 represented an enigma, as this type of cancer was rare and unusual in young women [16]. No obvious causal factors were originally identified. The resolution of this enigma was facilitated by the deceptively simple consideration of *in-utero *exposures among the cancer cases. A case-control study then implicated maternal diethylstilbestrol (DES) as the causal factor in subsequent development of vaginal adenocarcinoma in the exposed daughters [16]. Sometimes the resolution of an enigma is much more complex, as evidenced by the "Enigma machine" used by the German military to encode secrets during World War II. The basis for this enigma was the sheer number of possible internal connections within the machine, far surpassing the capability of any individual or small group to decode the information. This enigma was overcome only by the concerted efforts of many cryptanalysts working synergistically with Allied forces throughout the war.

Osteopathic medicine has its enigmas as well. An enigma integral to osteopathic principles involves the complex inter-relationships among the spinal cord, autonomic nervous system, and viscera. Louisa Burns performed animal research in this area in the early 1900s and was the first osteopathic investigator to establish and carry on a long-term research program [17]. J. Stedman Denslow moved the field of osteopathic research forward in the 1940s at the Kirksville College of Osteopathy and Surgery, where he also recruited many able investigators, including Irvin Korr [17]. However, the clinical applications of such research have materialized slowly over time and much remains to be explored at present. The preliminary results of case-control studies at the Osteopathic Research Center have shown significant associations between osteopathic palpatory findings and chronic diseases, including type 2 diabetes mellitus [18] and hypertension [19]. As with the Enigma machine, resolution of such osteopathic enigmas will require the concerted efforts of investigators dedicated to basic mechanistic research and its translation into clinical practice. Ominously, however, there appears to be a dearth of basic investigators within the colleges of osteopathic medicine to respond to this research challenge.

**Figure 4:**
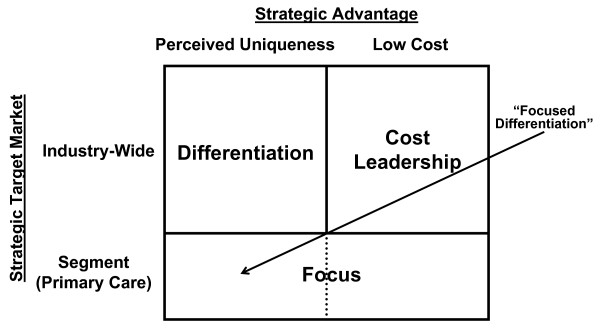
Generic strategies for competitive advantage according to dimensions of strategic advantage and strategic target market.

Osteopathic medicine faces another enigma at a different level – establishing a professional identity that is compatible with the demands and expectations of contemporary medical practice. In this regard, the osteopathic profession may do well to adopt a generic strategy aimed at achieving osteopathic "competitive advantage." As shown in Figure 4, the two-dimensional matrix used to select an appropriate strategy involves a consideration of osteopathic medicine's strategic advantage and its strategic target market [20].

Some considerations in making conventional business decisions involving the dimension of strategic advantage may not apply directly to the health care market in the United States. For example, the cost (i.e., price paid) for a health care service may be largely constrained by third-party payers who negotiate fixed fees. In such an environment, low cost leadership may not represent a viable strategy. Even in the classical market situation, in which costs are free to vary according to such factors as demand, cost leadership does not represent a strategy to be embraced by osteopathic physicians. Cost leadership implies large economies of scale that are generally not seen in osteopathic medical facilities and an "assembly-line" mentality that is incompatible with the osteopathic "hands-on" approach to patient care. Thus, it is clear that osteopathic medicine must adopt perceived uniqueness as its mechanism for strategic advantage.

Based on osteopathic principles and current specialty practice patterns, few would argue against focusing on primary care as the appropriate strategic target market segment for osteopathic physicians. The relatively small number of osteopathic physicians in non-primary care specialties [21] effectively precludes successful targeting of a health care industry-wide market and adoption of a pure differentiation strategy. Consequently, the osteopathic profession should adopt a "focused differentiation" approach to positioning itself and competing within the health care industry.

### Implementing a focused differentiation strategy for osteopathic medicine

Implementing a focused differentiation strategy will entail some challenges and potential risks for osteopathic medicine. From an osteopathic professional perspective, there are four basic requirements for implementing a focused differentiation strategy [20]: (1) a long tradition within the industry; (2) strong marketing abilities; (3) strong capacity in basic research; and (4) ability to attract highly skilled scientists, students, and creative people. Osteopathic medicine has an established position on the American medical landscape; however, it has failed to effectively market itself. Despite its potentially broad appeal through a primary care focus and high levels of satisfaction among established patients [22], only about half of the United States population claims to be aware of osteopathic medicine [23]. As commented above, there is a scarcity of basic scientists at the colleges of osteopathic medicine who are able and willing to provide the profession's much-needed research capacity. Appropriate incentives are needed to attract such scientists, to support their development and collaboration with clinical investigators, and to retain them over time. Additionally, the colleges of osteopathic medicine must seek to recruit highly-qualified students who truly believe and adhere to osteopathic philosophy. Two trends that make such student recruitment problematic, at least presently, are the accelerating rate of establishment of new colleges of osteopathic medicine and the increasing number of osteopathic physicians entering residency programs accredited by the Accreditation Council for Graduate Medical Education (ACGME). Assuming a fixed, limited number of highly qualified and desirable "osteopathically-oriented" applicants nationwide, expansion of the colleges of osteopathic medicine will generally lead to a dilution of such applicants with less optimal applicants. The training of larger numbers of osteopathic medical school graduates, particularly those of marginal osteopathic orientation, in ACGME-accredited primary care residency programs [24] may also have an inhibitory influence on osteopathic philosophy and practices, including OMT.

There are two potential risks for osteopathic medicine in implementing a focused differentiation strategy [20]: (1) differences between the market segments with respect to health care services diminish over time and the health care market as a whole converges; and (2) other health care providers find submarkets within primary care and "outfocus" osteopathic physicians. With advances in biotechnology and pharmaceuticals and an increasing proportion of allopathic physicians entering subspecialty residency programs [24], the risk of a convergence of the health care market appears very low for the foreseeable future. The risk of osteopathic physicians being "outfocused" is also low at present, but may be potentially greater in the long run. For example, if osteopathic physicians continue to focus on primary care, but progressively abandon their use of OMT (an aspect of their perceived uniqueness), then complementary and alternative medicine (CAM) practitioners, such as chiropractors, massage therapists, and acupuncturists, may become more prominent first-line providers for common primary care problems involving pain or musculoskeletal disorders. Similarly, integrative medicine physicians, CAM practitioners, and other non-physician clinicians may begin to encroach on traditional primary care boundaries. Overall, however, the risks for osteopathic medicine in implementing a focused differentiation strategy are low and are acceptable relative to the potential advantages to be derived from such a strategy. Failure to adopt a coherent strategy to develop and promote a professional identity will likely ensure that osteopathic medicine remains "stuck in the middle" – a poor strategic situation [20]. In response to this identity challenge, I recommend using the term "*omtology*" to refer to OMT-related research and scholarly activity. Similarly, derivative terms such as "*omtoepidemiology*," "*omtogenetics*," and "*omtovigilance*" may be used to refer to specific fields of research within the realm of OMT.

## Conclusion

The question of OMT efficacy is not the elephant in the room. While much osteopathic research – mechanistic, translational, and clinical – remains to be performed, the clinical trials involving low back pain, taken together in a meta-analysis, have provided evidence of OMT efficacy. More importantly, the role of OMT and the emphasis on primary care have not been adequately reconciled in developing a professional identity for osteopathic medicine. A focused differentiation strategy that entails both of these aspects should be adopted by osteopathic medicine to help develop and promote its professional identity.

## Competing interests

JCL is Editor-in-Chief of *Osteopathic Medicine and Primary Care*. He was not involved in the review of this manuscript or in the editorial decision with respect to its suitability for publication.
